# Modified interpretation criteria significantly improve performance of commercially available confirmatory assays for the serodiagnosis of Lyme borreliosis: a case-control study with clinically defined serum samples

**DOI:** 10.1007/s10096-018-03455-1

**Published:** 2019-02-04

**Authors:** Ulrike Hauser

**Affiliations:** SYNLAB MVZ Augsburg GmbH, Gubener Straße 39, 86156 Augsburg, Germany

**Keywords:** Lyme borreliosis, Confirmatory test, Immunoblot, Multiplex, Chip, Spot immunoassay

## Abstract

Case-control study for the evaluation of innovative test formats for second-tier testing for the serodiagnosis of Lyme borreliosis (LB). A head-to-head comparison was performed with the test systems ViraStripe, SeraSpot, ViraChip, and recomBead. Serum samples from 62 patients (21 erythema migrans, 33 Lyme neuroborreliosis, 8 late LB) and 91 controls (including 29 potentially cross-reacting sera) were tested. For ViraChip and recomBead, optimised interpretation criteria were developed for both IgG and IgM. The most important modification for the proposed interpretation criteria for ViraChip is the interpretation of strong (> 2.5-fold above cutoff) singular IgG reactions against VlsE as positive. This significantly improves sensitivity (32 to 85%, *p* < 0.0001) without significant changes in specificity (borderline reactions interpreted as negative). By application of our modified rules, specificity of ViraChip IgM is significantly increased (89 to 97%, *p* < 0.05; borderline results included to negatives), and sensitivities of recomBead IgG and IgM are also significantly improved (69 to 87%, *p* < 0.01, and 57 to 74%, *p* < 0.01, respectively; borderline results included to positives). Further improvement of sensitivity by the rating of strong singular IgG reactions against VlsE as positive can also be shown for recomBead. IgG/IgM result combinations must be interpreted as a function of the assumed disease stage, and the best combinations differ for the various assays. Application of our proposed interpretation criteria significantly improve the discriminatory abilities of two assays; however, this must be confirmed with other data sets. Recommendations from Scientific Societies should be updated as may be necessary.

## Introduction

Lyme borreliosis (LB) is the most frequent tick-borne disease in the northern hemisphere and is caused by different species of the *Borrelia burgdorferi* sensu lato group (*B. burgdorferi* s.l.) [1, 2]. In Europe, at present, *B. burgdorferi* sensu stricto (*B. burgdorferi* s.s.), *B. garinii*, *B. afzelii*, *B. spielmanii*, and *B. bavariensis* are considered pathogenic for humans [3]. The disease comprises various manifestations involving the skin, the nervous system, the joints, and, less frequently, the heart or the eyes [4]. The diagnosis is basically clinical and is supported by laboratory findings, whereas antibody testing is the easiest and most widely used method [1–5]. However, both clinical symptoms can be atypical and difficult to diagnose as well as serology is hampered by various pitfalls and thus can lead to misinterpretations. The humoral immune response develops slowly and testing can still be negative in the early stage. In patients with prompt treatment of early disease, an increase of antibodies and especially the Ig-class switch to IgG can be missing. On the other hand, IgG and even IgM antibodies can persist for years after symptoms have resolved [1, 3]. Unspecific cross-reactions most notably occurring in IgM tests can be observed under various conditions, e.g. acute infections with Epstein–Barr virus (EBV), cytomegalovirus (CMV), or other viruses or *Mycoplasma pneumoniae* as well as autoimmune diseases [3, 6, 7]. The heterogeneity of *Borrelia* strains and the successive appearance of antibodies against different antigens in the course of the infection further contribute to complexity [1, 8–12]. These difficulties also lead to a low diagnostic accuracy of serological tests [13].

In Europe, as well as in the USA, in most countries a two-step approach is recommended for serological testing [3, 14, 15]. The first step shall be a highly sensitive ELISA or similar test format, and positive or borderline results shall be retested with a highly specific blot. The aim of this procedure is an increase in the overall performance in terms of both sensitivity and specificity and thus, predictive values.

Commercially available assays use a variety of antigen compositions including also antigen homologues derived from different strains. The most important antigens are p100/83, p58, p41 (e.g. internal fragments), p39 (BmpA), OspC, DbpA (Osp17), VlsE [2, 3, 8, 10, 16]. For whole cell Western blots with *B. afzelii* strain PKo and also for immunoblots with recombinant antigens representing this antigen composition (e.g., ViraStripe) p43, p30, p21, and p14 are important as well [3, 8].

In an attempt to facilitate workflow in high throughput laboratories, innovative test formats like multiplex bead or spot array technologies have been developed to replace strip immunoassays.

However, up to now, the introduction of these assays into routine diagnostics is deferred, since only limited evaluation data is available for these assays [17, 18] and especially, studies using clinically defined samples are rare [19, 20].

The initial aim of our preliminary study was an evaluation of a bead-based multiplex assay (recomBead, MIKROGEN) and two spot array tests (ViraChip, Viramed and SeraSpot, Seramun) by a head to head comparison with the so far performed line immunoassay (ViraStripe, Viramed) prior to the introduction of one of these test systems into routine diagnostics. Therefore, panels of selected sera from patients with LB meeting European case definitions [4], as well as a control group including samples from healthy persons and potentially cross-reacting sera were used. After all sera were tested, sensitivities and specificities were assessed. By the analysis of raw data, it was realised that modification of the interpretation criteria given by the manufacturers of two of the assays might lead to considerably improved performance.

## Materials and methods

### Samples

For the determination of sensitivities, only remnant serum samples from patients with symptoms conform to European case definitions for LB [4] were tested. The three groups comprised 21 patients with erythema migrans (EM), 33 patients with acute Lyme neuroborreliosis (LNB), and 8 patients with late-stage LB. Clinical data was documented from counselling and discussion of reports with clinicians by telephone during routine diagnostics. Only patients with skin lesions typically for EM as designated by the clinicians were selected. However, usually, only patients with at least borderline reactivity in the screening are discussed and thus could be included. (Screening ELISA: Enzygnost Lyme link VlsE/IgG and Enzygnost Borreliosis/IgM, Siemens, Germany) Serological preselection was not intended, but could not generally be avoided in this group. One 6-year-old girl with a facial palsy had a lymphocytic pleocytosis in the cerebrospinal fluid (CSF) and was reactive for *Borrelia* IgG in CSF, however, no CSF/serum IgG antibody index could be calculated due to the negative *Borrelia* IgG in serum. All other patients with acute neuroborreliosis showed lymphocytic pleocytosis in CSF, CSF/serum *Borrelia* IgG antibody indices > = 2.0, and typical clinical symptoms [21]. The group of patients with late LB comprised seven patients with Lyme arthritis (typical clinical signs, high levels of specific IgG, differential diagnoses excluded) and one patient with acrodermatitis chronica atrophicans (ACA) diagnosed by a dermatologist.

The control group for the determination of specificities comprised 93 samples. Fifty-seven sera were obtained from healthy persons (mostly staff), who negated tick bites, erythemas, neurological symptoms, or joint disorders. A total of 31 sera from patients with diseases potentially leading to cross-reactions (7 with anti-nuclear antibody titres greater or equal to 1000, 4 with syphilis, 1 with a CMV, and 9 with EBV primary infections, 10 with rheumatoid factor greater than 50 IU/ml) and 5 sera from patients with unspecific symptoms not consistent with LB were also included.

After the classification of patients and controls in the respective diagnosis groups, sera were made anonymous by the assignment of new sample identification numbers not connected to patient identity.

### Test systems

The following assays were performed: routine assay: *Borrelia* ViraStripe IgG, IgM (VIRAMED Biotech AG, Planegg, Germany); comparison assays: recomBead *Borrelia* IgG, IgM 2.0 (MIKROGEN GmbH, Neuried, Germany), SeraSpot Anti-*Borrelia*-10 IgG, IgM (Seramun Diagnostica GmbH, Heidesee, Germany), and *Borrelia* ViraChip IgG, IgM (VIRAMED Biotech AG). Devices and assessment systems were provided by the respective manufacturers. Sera were tested and interpreted according to the manufacturers’ instructions; however, interpretation of the line immunoassay ViraStripe was slightly modified according to in-house criteria including a borderline zone for both IgG and IgM.

The test principle of recomBead *Borrelia* IgG, IgM 2.0 respective is based on the Luminex system: highly purified, recombinant *Borrelia burgdorferi* s.l. antigens (OspA, OspC, p100, VlsE, p39, p58, DbpA) are applied separately to different microparticles (beads) with differing fluorescence codings. Antibodies against individual antigens are recorded separately from each other in one solution. The average fluorescence intensities of all individual antigen/antibody reactivities of each sample are compared with the reactivities of an incubation control and a cutoff index (COI) is calculated via a batch-dependent limit value by the software recomQuant. The reactivity against an individual antigen is interpreted as negative for a COI < 0.67, borderline if 0.67 < = COI < 1.00, and positive for COI > = 1.00. The test results are obtained by a point evaluation system for the individual reactions. Therefore, the corresponding point values in the case of borderline and positive results for individual antigens are added. For the development of optimised criteria, only the point evaluation system was modified (see below).

SeraSpot Anti-*Borrelia*-10 IgG, IgM respective is a spot immunoassay based on the use of *Borrelia* antigens printed in an array arrangement (spot array) directly on the bottom of each well of a 96-well microtiter plate. The following recombinant antigens are used for the individual spots: VlsE (*B. afzelii*), p39 (*B. afzelii*), p58 (*B. garinii*), p100 (*B. afzelii*), OspC (*B. afzelii*), OspC (*B. garinii*), OspC (*B. burgdorferi* s.s.), DbpA (*B. afzelii*), DbpA (*B. garinii*), and DbpA (*B. burgdorferi* s.s.). After test processing, wells with spots consisting of coloured immune complexes are scanned and interpreted automatically by the software Seramun SpotSight scan.

*Borrelia* ViraChip IgG, IgM respective is an immunoblot in a microarray format carrying highly purified specific native antigens from *B. afzelii* (strain Pko) and *B. burgdorferi* s.s. as well as recombinant VlsE. Microarrays are spotted onto nitrocellulose and fixed at the bottom of each well in a standard microtiter plate. The analyte spots of the IgG test serve to detect antibodies against p83, p58, p43, p39, p30, p21, OspC, DbpA/Osp17, p14, and VlsE, whereas the arrays for IgM testing contain p41, p39, OspC, Osp17, and VlsE. Prior to scanning and automatic interpretation, the microtiter plates must be dried. The measured mean intensity of the calibrator controls is multiplied by the lot specific factor for each antigen. The resulting value is used as the cutoff for the assessment of the respective analyte spots (three spots per antigen). Triplets with a mean intensity equal to or higher than the cutoff (i.e. COI > = 100) are considered ‘distinct’ and are incorporated in the interpretation. For our modification of the interpretation criteria calculations with these COIs were performed.

### Processing

Testing of ViraStripe and recomBead was performed in August 2014, ViraChip and SeraSpot were tested with the same sera in July 2017. All tests were performed blindly. Sera were stored continuously at − 20 °C in the meantime. For the determination of the overall sensitivities, all diagnosis groups were included for the IgG assays, whereas for the IgM tests only data from patients with EM or LNB were evaluated. IgM tests were not performed for late LB due to lacking clinical relevance [3, 5].

Sera with discordant results between ViraStripe and recomBead were retested with recomBead for confirmation of the results. All the sera were tested at least twice with different lots of ViraChip. Samples not reacting concordant were tested again with a third lot. Sera with discordant reactivity between ViraChip and SeraSpot were tested also with a different lot of SeraSpot. Reactivity was considered borderline for samples that reacted positive once and negative once in runs with different lots of SeraSpot.

### Development of optimised interpretation criteria

Tables with raw data of measured values for each single antigen for all tested sera were analysed for patterns typical for true positive and false positive results. For both recomBead and ViraChip, COIs were taken for this analysis.

Sensitivities and specificities for a variety of presumed criteria were calculated and the criteria with the best discriminatory abilities were chosen. Since the optimised criteria for ViraChip focus on the uprate of singular strong IgG reactivity with VlsE, further calculations were carried out also for recomBead and SeraSpot with the subset of sera with singular VlsE-IgG reactivity.

The ‘optimised criteria’ for recomBead were developed in 2014 basing on the two-band criterion for a positive IgG blot recommended by the German Society for Hygiene and Medical Microbiology [22] (updated in 2017 [3]) and were adopted by the manufacturer in June 2016. On the other hand, the ‘suggested criteria’ were developed much later in 2018 by calculations based on our data only.

### Notation of assays and interpretation rules

In the following text, the term ‘assay’ is used for both assays from different manufacturers as well as for different interpretation rules for the same test. The following notation is used throughout the subsequent text: VC-orig: ViraChip with original interpretation criteria according to the manufacturer; VC-ih: ViraChip assessed with in house criteria; RB-orig: recomBead with original interpretation criteria according to the manufacturer prior to June 2016; RB-opt: recomBead with optimised criteria developed in 2014, adopted by the manufacturer in June 2016; RB-VlsE: recomBead with criteria suggested in 2018 (for IgG only).

### Analysis of combined test results of both IgG and IgM tests (see Table 3 for exemplification)

Frequencies of all possible IgG/IgM result combinations were compared for patients and controls. The following acronyms were used: GpMp: IgG positive and IgM positive; GpMb: IgG positive and IgM borderline; GpMn: IgG positive and IgM negative; GbMp: IgG borderline and IgM positive; GbMb: IgG borderline and IgM borderline; GbMn: IgG borderline and IgM negative; GnMp: IgG negative and IgM positive; GnMb: IgG negative and IgM borderline; GnMn: IgG negative and IgM negative; and GpMne: IgG positive, IgM not evaluable (due to invalid assay). For further exploratory analysis, simple arbitrary rules and calculations were applied to describe discriminatory abilities of the different assays. Separate analyses were performed for EM and LNB. The results of the most important combinations for the detection of cases were added. In doing so, specificities equal or greater than 95% were required. Therefore combinations with more than 4 controls (*n* = 91) were not included in the counts. Two sums were calculated for each diagnosis group. The first sum denominated ‘common trunk’ consists of all combinations applicable for all assays. ‘Common trunks’ differ for diagnosis groups but not for assays. For the second sum for ‘best discrimination’ for each assay, the ‘common trunk’ was individually complemented with further combinations adding to sensitivity (without loss of specificity below 95%, however). Combinations included in ‘best discrimination’ are boxed in the table.

### Statistics

McNemar’s test was used for pairwise comparisons of sensitivities and specificities.

Chi-square test of independence was used to analyse sensitivities and specificities of the seven assays in the combined IgG/IgM evaluation. All analyses were performed two-sided, and *p* values < 0.05 were considered to be significant.

AUCs (area under the receiver operating characteristics (ROC)-curve) were calculated for comparisons of diagnostic performance since standardisation of specificities was not possible.

GraphPad Prism version 6.01 was used for all calculations.

## Results

Two sera of the potentially cross-reacting group were excluded from the analysis due to their strong reactivity in all IgG and all IgG and IgM tests, respectively. For simplification, the control group is presented without differentiation in healthy persons, cross-reacting sera, or patients with unspecific symptoms.

After the determination of sensitivities and specificities by the assessment with the original interpretation criteria, optimised criteria were developed for both ViraChip and recomBead.

In Table 1, original criteria are opposed to modified criteria for ViraChip. Sensitivity of the IgG assay was significantly increased by rating of singular VlsE triplets as positive if reactivity is at least 2.5-fold above the cutoff. This decision threshold was set to achieve the highest possible sensitivity while maintaining a specificity of at least 95%. Singular OspC triplets are evaluated borderline. Specificity of the IgM assay was improved by a more differentiated rating of the individual reactions.

**Table 1:** Modification of interpretation criteria for ViraChip

Assay	Result	Criteria
(A) Original criteria according to manufacturer (VC-orig)		
IgG	Positive	At least two triplets with intensity > = 100 out of: p83, p58, p43, p39, p30, OspC, p21, Osp17/DbpA, p14, or VlsE
	Borderline	VlsE triplet with intensity > = 100
	Negative	One or zero triplets with intensity > = 100 (exception: singular VlsE triplet)
IgM	Positive	At least one triplet with intensity > = 100 out of: p41, p39, OspC, Osp17, or VlsE
	Negative	No triplet with intensity > = 100
B) Modified in house criteria (VC-ih)		
IgG	Positive	At least two triplets with intensity > = 100 out of: p83, p58, p43, p39, p30, OspC, p21, Osp17/DbpA, p14, or VlsE or VlsE triplet with intensity > = 250
	Borderline	VlsE triplet with intensity 100–249 or OspC triplet with intensity > = 100
	Negative	One or zero triplets with intensity > = 100 (exception: singular VlsE or OspC triplet)
IgM	Positive	At least one triplet with intensity > = 140 out of: p41, p39, Osp17, or VlsE or OspC triplet with intensity > = 120 or at least two triplets with intensity > = 100 out of: p41, p39, OspC, Osp17, or VlsE or one triplet with intensity > = 100 and at least one other triplet with intensity > = 80
	Borderline	One triplet with intensity 100–139 out of: p41, p39, Osp17, or VlsE (and no other triplet with intensity > = 80) or OspC triplet with intensity 100–119 (and no other triplet with intensity > = 80)
	Negative	No triplet with intensity > = 100

Table 2 shows original (A; RB-orig ) and optimised criteria (B; RB-opt) for recomBead. In contrast to the original criteria, for the IgG assay, reactions with VlsE as well as borderline reactions are relatively uprated to increase sensitivity. Sensitivity of recomBead IgM was also improved due to the uprate of borderline reactions. The additional analysis of IgG data in March 2018 lead to the suggested criteria RB-VlsE (C). The most important modification is the interpretation of singular VlsE-IgG reactions at least 2.5-fold above the cutoff as positive. However, any other threshold between 1.8 and 2.7 would not have changed sensitivity and specificity in our data set, since there were no samples with reactivities in this range.

For SeraSpot, no improvement could be demonstrated by a modified assessment of singular VlsE-IgG reactions.

**Table 2:** Modification of interpretation criteria for recomBead

(A) Original criteria according to manufacturer (RB-orig)		
(a) Point assessment for reactions with antigens		
Assay	Individual reaction	Points
IgG	Each positive reaction with any antigen	4
	Each borderline reaction with any antigen	1
IgM	Each positive reaction with any antigen except OspC	4
	Positive reaction with OspC	8
	Each borderline reaction with any antigen	1
(b) Test interpretation		
Evaluation	Sum of points IgG	Sum of points IgM
Negative	0–4	0–4
Borderline	5–7	5–7
Positive	> 7	> 7
(B) Proposed optimised criteria 2014, adopted by the manufacturer starting from June 2016 (RB-opt)		
(a) Point assessment for reactions with antigens		
Assay	Individual reaction	Points
IgG	Each positive reaction with any antigen except VlsE	2
	Positive reaction with VlsE	3
	Each borderline reaction with any antigen	1
IgM	Each positive reaction with any antigen except OspC	2
	Positive reaction with OspC	4
	Each borderline reaction with any antigen	1
(b) Test interpretation		
Evaluation	Sum of points IgG	Sum of points IgM
Negative	< 3	< 2
Borderline	3	2–3
Positive	> 3	> 3
(C) Suggested criteria 2018 (RB-VlsE)^a^		
(a) Point assessment for reactions with antigens		
Assay	Individual reaction	Points
IgG	Each positive reaction with any antigen except VlsE	2
	Positive reaction with VlsE with COI > = 2.5	4
	Positive reaction with VlsE; 1.0 < = COI < 2.5	3
	Each borderline reaction with any antigen except VlsE	1
	Borderline reaction with VlsE	2

^a^Points for IgM and sum of points for test interpretation for both IgG and IgM see (B) Proposed optimised criteria 2014 (RB-opt)

**Fig. 1:**
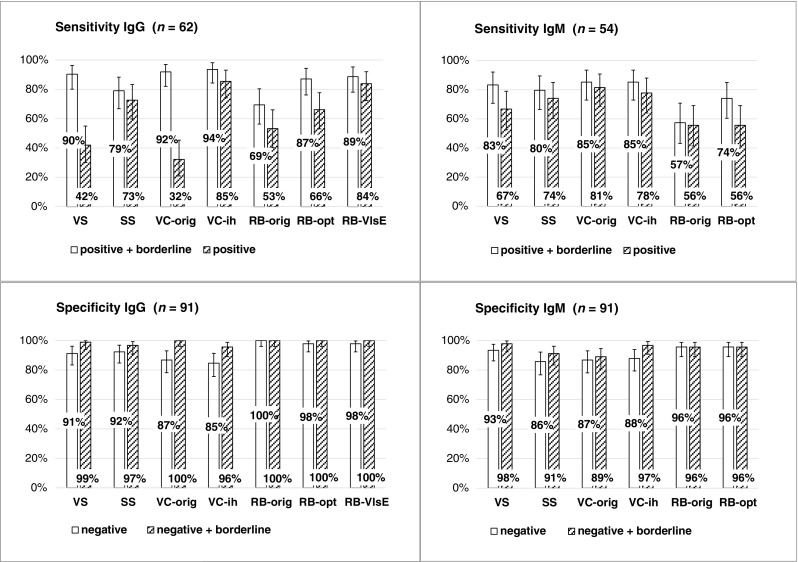
Performance of different assays including assessment with modified interpretation criteria. VS, ViraStripe; SS, SeraSpot; VC-orig, ViraChip with original interpretation criteria according to the manufacturer; VC-ih, ViraChip assessed with in house criteria; RB-orig, recomBead with original interpretation criteria according to the manufacturer prior to June 2016; RB-opt, recomBead with optimised criteria developed in 2014, adopted by the manufacturer in June 2016; RB-VlsE, recomBead with criteria suggested in 2018 (for IgG only). White bars: borderline results interpreted as positive. Shaded bars: borderline results interpreted as negative. Error bars: 95% confidence intervals

Figure 1 presents the performance of all assays with the application of original interpretation criteria as well as modified criteria. Calculations of sensitivities and specificities were done in duplicate (i) under the assumption that borderline reactions are interpreted as positive (white bars) and (ii) if borderline reactions are interpreted as negative (shaded bars).

For IgG of both ViraStripe and VC-orig, the differences of sensitivities between these two evaluations are significant due to the high proportion of borderline reactions (ViraStripe 90 versus 42%: *p* < 0.0001; VC-orig 92 versus 32%: *p* < 0.0001).

The evaluation with borderline results included to negatives (shaded bars) showed that the sensitivity of ViraChip IgG was improved from 32 to 85% (*p* < 0.0001) by the use of our in-house criteria and this was linked to a moderate deficit in specificity (100 versus 96%; *p* > 0.05) only. Sensitivity of recomBead IgG was first improved from 53 to 66% (RB-orig versus RB-opt; *p* < 0.05), and then from 66 to 84% (RB-opt versus RB-VlsE; *p* < 0.01) without changes of specificity (100%). Specificity of ViraChip IgM could be raised from 89 to 97% (*p* < 0.05) with only a minor loss of sensitivity (81 versus 78%; *p* > 0.05).

If borderline results are included to positives (white bars), for recomBead IgG, sensitivity was improved from 69 to 87 or 89% (*p* < 0.01) for RB-opt and RB-VlsE, respectively, and this was linked to an insignificant deficit in specificity (100 versus 98%) only. For recomBead IgM, sensitivity could be increased from 57 to 74% (*p* < 0.01) without deficit of specificity (96%).

Of the IgG assays, only recomBead (all interpretation rules) shows specificities above 95% for both assessments. For all other IgG tests, borderline reactions must be interpreted as negative to achieve a specificity of at least 95%. recomBead has the highest specificity for IgM also (96% for both evaluations and both interpretation rules). On the other hand, specificities of two other IgM assays are lower than 95% even if borderline reactions are interpreted as negative (SeraSpot 91%, VC-orig 89%).

The eight sera from patients with late LB reacted positive with all IgG assays.

Table 3 shows the frequencies of combined IgG and IgM results for patients and controls. Separate analyses were performed for EM and LNB. Two groups of combinations were summarised: For EM, the ‘common trunk’ comprises GpMp, GpMb, GbMp and GbMb, i.e. sera with both positive or borderline IgG and IgM reactivity. For LNB, it consists of GpMp, GpMb and GpMn, i.e. any combination with a positive IgG. Sensitivities of the ‘common trunks’ differed significantly (EM *p* < 0.01; LNB *p* < 0.0001), whereas the ‘best discriminations packages’ (summary of the boxed cells in the table) performed in a comparable range (EM 71–90%; LNB 88–100%) with an exception for SeraSpot for EM with a sensitivity of 48% only (EM *p* > 0.05, if SeraSpot was excluded; LNB *p* > 0.05). On the other hand, SeraSpot performed very well for LNB (GpMp 25/33).

**Table 3:**
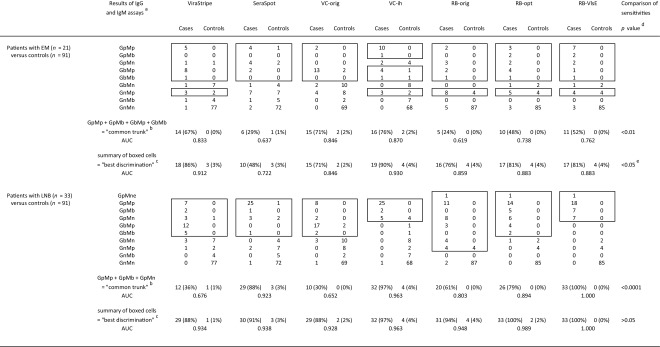
Combined evaluation of IgG and IgM assays in different stages of LB (absolute frequencies)

AUC, area under the ROC-curve

^a^GpMp: IgG positive and IgM positive; GpMb: IgG positive and IgM borderline; GpMn: IgG positive and IgM negative; GbMp: IgG borderline and IgM positive; GbMb: IgG borderline and IgM borderline; GbMn: IgG borderline and IgM negative; GnMp: IgG negative and IgM positive; GnMb: IgG negative and IgM borderline; GnMn: IgG negative and IgM negative; GpMne: IgG positive, IgM not evaluable (due to invalid assay)

^b^The summary of the results of the most important combinations in the respective diagnosis group was referred to as ‘common trunk’. ‘Common trunks’ differ for diagnosis groups but not for assays

^c^Results of combinations detecting at least as many patients as controls were boxed. Specificity should be at least 95%. The sum of all frequencies in the boxed cells is given in the row ‘best discrimination’. ‘Best discriminations’ differ for both diagnosis groups and assays

^d^Sensitivities of the different assay were analysed by chi-square test

^e^*p* > 0.05, if SeraSpot was excluded

For EM, the combinations included for the ‘best discrimination’ differed in respect to the consideration of GpMn and GnMp. GnMp could not be included for SeraSpot and VC-orig due to unspecific reactivity (up to 9% of the controls). For LNB, for VC-ih and for RB-VlsE, the ‘best discrimination’ equals the ‘common trunk’. For the other assays, at least GbMp and GbMb had to be complemented for ‘best discrimination’. The highest AUCs were achieved for VC-ih for EM and with RB-VlsE for LNB.

## Discussion

In this study significant improvement of performance of ViraChip as well as recomBead through the application of modified interpretation criteria could be demonstrated (Fig. 1). Most notably, the increase of sensitivity of the IgG assay was extremely significant for ViraChip (32 versus 85%) without significant change of specificity. Our optimised interpretation criteria for recomBead were adopted by the manufacturer in June 2016. However, as compared to these rules (RB-opt) another increase of sensitivity for IgG from 66 to 84% could be shown by the application of RB-VlsE.

Since the number of tested sera was rather low in this preliminary study, all modifications were done with caution, e.g. only the grayzone was enlarged for VC-ih IgM and RB-opt IgM. The decision thresholds for the rating of singular VlsE-IgG as positive were also set in favour of high specificity for both VC-ih and RB-VlsE. Setting this threshold at 2.0 instead of 2.5-fold above the cutoff for ViraChip would lead to a gain of sensitivity of 3% (2/62 samples) and a loss of specificity of 1% (1/91 samples) in our data set.

### Borderline results

The classification ‘borderline’ plays a considerable role for the evaluations in this study. In the case of borderline results, a follow-up sample should be tested if necessary [3, 5]. Furthermore, however, the knowledge of the performance data of the assays enables the interpretation of borderline rather as positive (e.g. recomBead IgG and IgM) or negative (e.g. VC-ih IgG). Besides this, classification as borderline can help to ‘smoothen’ the irritating effects of imprecision. In routine diagnostics, this effect is especially obvious if follow-up sera with reactivities near a classification threshold are tested.

### IgG/IgM combinations

The discriminatory abilities of the different assays were analysed further by a combined evaluation of both IgG and IgM results in different stages of LB (Table 3). The patients with EM serve as a model for the early local stage, whereas LNB should represent early disseminated disease. The intention of this exploratory analysis was to find out for each assay which combination of test results rather support a clinical suspicion and which combinations rather argue against it. Of course, this analysis is strongly influenced by the (pre)selection of the tested sera; most notably, the distribution of the controls is critical. Specificities equal or greater than 95% were required for the sums ‘common trunk’ as well as ‘best discrimination’ since second-tier tests were evaluated and in daily routine, LB must more often be ruled out than confirmed.

VC-ih and RB-VlsE performed the best, but the application of individually modified ‘best discrimination packages’ showed no significant differences in sensitivities (and specificities) of all tests and AUCs in similar ranges (exception: SeraSpot for EM). This contrasting juxtaposition with arbitrary determinations was intended to demonstrate the ambiguous meaning of certain constellations.

However, particularly this data is preliminary since the number of cases and controls is too small for such a differentiated analysis. The descriptive format of this analysis was chosen to reflect the routine situation showing combinations of results in the familiar format (What could this constellation mean?).

For EM, GpMn was not included for ‘best discrimination’ for VC-ih. This reflects the higher background IgG reactivity and the higher IgM specificity of our in-house criteria (see Fig. 1).

### ‘IgM only’

The most critical combination is GnMp, i.e. isolated positive IgM with negative IgG. As demonstrated in the evaluations for both EM and LNB and as is widely discussed in the literature [3, 5–7, 23], this can mean ‘anything’: It could be the beginning of the serological detectability of an acute infection or unspecific or polyclonal reactivity due to another aetiology. In a routine setting, follow-up controls are mandatory in most cases prior to the beginning of an antibiotic therapy.

### Diagnostic scores

For the combined evaluation of tests, Dessau et al. constructed diagnostic scores by logistic regression of data from patients with LNB as well as controls [19, 24]. This is a good statistical approach, but for different stages of LB, possibly different scores, e.g. with a different weighting of IgM would be necessary. For the patients with LNB, for one of the compared assays (IDEIA, Oxoid) a complementation of IgG and IgM could be demonstrated as well [24]. In the evaluation of recomBead, VlsE also turned out to be by far the most important antigen for IgG detection, however, a combination with other antigens lead to a slight further increase of discriminatory ability [19]. In comparison to the assessment provided by the manufacturer, improvement of discriminatory power through the application of the diagnostic scores could be demonstrated as well. In the comparison of Danish and Swedish samples, considerable different background reactivity leading to remarkably different cutoffs was shown [19].

### VlsE

In our study, the significant improvement of sensitivity of both ViraChip and recomBead IgG was due to the rating of strong isolated reactivities with VlsE as positive. The original interpretation criteria by the manufacturers follow the recommendations by the German Society for Hygiene and Medical Microbiology (DGHM) for the interpretation of *Borrelia* IgG immunoblots, which require at least two bands for a positive result [3]. However, this two-band criterion was originally established before VlsE as a very powerful diagnostic antigen was known [8, 22, 25]. The introduction of VlsE for recombinant immunoblots lead to a significant improvement, but apparently, the two-band criterion was still necessary to achieve adequate specificity [10, 16]. However, in these studies, only one cutoff was used. The analysis of data from testing with recomBead by Dessau et al. showed a high sensitivity of VlsE-IgG alone even with high cutoffs adapted to the background seroprevalence detected by the Swedish control samples [19]. In the USA, two-tiered testing with the replacement of blots by an immunoassay using only a C6-peptid derived from VlsE is discussed [26]. Optimisation of VlsE-IgG reactivity would probably improve sensitivity of SeraSpot for EM.

### Precision

The different rating of reactivities as a function of signal strength requires a second (higher) cutoff and sufficient precision in this measuring range. Even if not designed for quantitative analyses, the new test systems might meet this requirement; however, this should be studied further. All of them are processed and analysed automatically, and subjective visual reading is not even possible. In our evaluation, inter assay imprecision of VlsE-IgG was acceptable for the three tested lots of ViraChip IgG (coefficient of variation: mean 15%, range 9–21%) and good for all runs within the same lot of recomBead IgG (coefficient of variation: mean 4%, range 0–14%). This analysis included COIs between 0.5 and 3.0 or 50 and 300, respectively (detailed data not shown). Perhaps, a second calibrator for VlsE could be helpful.

### Selection of sera from patients with LNB and EM

A prerequisite for an evaluation of diagnostic assays in a case-control format is the availability of well-defined samples. Our panel from LB patients comprises mainly patients with LNB, as these cases can be unambiguously identified by laboratory findings. Since EM is primarily a clinical diagnosis, most patients are not tested serologically, and the accessibility of sera from typical cases is restricted. However, EM is by far the most frequent manifestation of LB, and therefore should be represented adequately in the test panel. Atypical manifestations of EM constitute a considerable challenge for clinicians. Some authors do not recommend serological testing of these cases [4]; nevertheless, the guideline of the German Dermatology Society recommends a clarification through a serological test as the first step of laboratory diagnostics [27]. The guideline states ‘only if the serological test is negative and the clinical suspicion remains, direct cultural or molecular-biological detection from biopsy material shall be used for clarification’ [27]. (Unfortunately, PCR testing of skin biopsies is not reimbursed by compulsory insurance in Germany.) Our findings suggest that this should be enlarged to all IgG/IgM result combinations not included in the ‘common trunk’ for EM. Furthermore, immune response in early infection is typically restricted to VlsE, OspC and p41.

### Late LB

Only eight sera from patients with late LB were included in our analysis as these sera were expected to show clear IgG reactivity. All of them showed IgG reactivity with various antigens. ViraStripe and ViraChip are based on the same antigen panel. However, for ViraStripe antigens are adjusted to resemble whole cell Western blots with *B. afzelii* strain PKo [3, 8], whereas for ViraChip adjustment of individual antigens was optimised for high sensitivity in early stages of infection and thus, antibodies usually occurring later on in the disease, e.g. against p83, p58, p39 or Osp17/DbpA are detected less frequently and less intense. From routine testing, it is known that sera from patients with *Borrelia* infections existing at least for months or reinfections show a typical broad IgG band pattern when tested with ViraStripe (see also [8, 12]). With ViraChip, this is by far less distinctive and the differentiation between stages of infection (e.g. acute versus existing at least for months) is less clear. If future assay development is orientated only on discrimination between positive and negative (e.g. by regression analyses), this additional information about antibody patterns might get lost.

### Control group

The control group should represent the challenges of the routine situations; consistency is critical and certain single samples can turn the balance. Therefore, high sample numbers are important. For the evaluation of IgG/IgM combinations, we required a specificity of at least 95%. Another approach would have been to intend a discrimination between background reactivity in the local population and active disease, however, this is also problematic to achieve. As far as possible, persons with suspicion of present or passed LB or noticed tick bites were not included in the group of healthy persons or into the group of patients with unspecific symptoms not consistent with LB. However, asymptomatic or passed infections with *Borrelia burgdorferi* s.l. can never be completely ruled out in this group. In some cases, differentiation between unspecific IgG reactivity and past infection is not possible. The potentially cross-reacting sera were collected by laboratory findings only and the history and present or past symptoms of these patients could not be taken into account. Two sera of this group were excluded from the analysis due to a strong reactivity in all IgG and all IgG and IgM tests, respectively. Ten percent of the sera of the total control group were taken from patients with acute EBV or CMV infections. For the evaluation of IgM tests, this means even a higher challenge than in the routine situation.

### Strength and limitations

The strength of our study is the head-to-head comparison of innovative test formats in a case-control design as there is almost no such data in the literature. The main limitation is the low number of samples, especially in the control group. (Initially, it was intended as an in-house orientation concerning assays already CE approved prior to the introduction of one of them into routine diagnostics.) Another limitation of our study is a certain preselection of sera due to positive or borderline IgG or IgM results with the screening ELISA (Enzygnost Lyme link VlsE/IgG and Enzygnost Borreliosis/IgM, Siemens, Germany). Our comparison was meant as an evaluation of the second step of the recommended two-tier testing and thus, included confirmatory assays only. As a matter of course, determined sensitivities in the group of patients with EM are higher as anticipated for unselected cases [8]. This does not seem to be a major problem, however, since the purpose of the study was a head-to-head comparison of different methods under the same conditions.

### Perspectives

Further studies should be performed with larger data sets including also different stages of LB to confirm and refine our proposals. For the interpretation of singular VlsE-IgG as positive, appropriate cutoffs should be determined through ROC analyses. If two cutoffs might be used in the future, reliable inter-lot precision must be assured for both.

Probably, fewer antigens would be sufficient to achieve high discriminatory power. Application of regression methods can weigh the role of the different antigens [19]; however, testing of sera from patients with different stages of LB is necessary [28].

The development of test systems by commercial manufacturers is crucially hampered due to the restricted access to clinically defined sera. In the USA, a collection of well-defined samples is available from the CDC [29]. The establishment of such a panel from European patients would be very beneficial. Furthermore, the samples should be available not only for approval studies but also for early stages of test development.

## Conclusion

Our preliminary study shows the potential for a significant improvement of innovative commercially available confirmatory tests. For the interpretation of IgG assays, a combination of the established rule ‘reactivity against at least two antigens’ (two-bands criterion) with the rating of isolated strong reactivity against VlsE as positive is favourable. However, further analyses with different and larger data sets are necessary. If our proposals could be verified, the specifications for confirmatory tests from the German Society for Hygiene and Medical Microbiology (DGHM) should be updated.
